# Multi-scale detection of rate and variance changes in neuronal spike trains

**DOI:** 10.1186/1471-2202-16-S1-P217

**Published:** 2015-12-18

**Authors:** Stefan Albert, Michael Messer, Brian Rummell, Torfi Sigurdsson, Gaby Schneider

**Affiliations:** 1 Institute of Mathematics, Goethe-University, Frankfurt am Main, Germany; 2 Institute of Neurophysiology, Goethe-University, Frankfurt am Main, Germany

## 

Neuronal spike trains can show variability with respect to process parameters such as the rate or variability of inter spike intervals. These changes can occur on fast and slow time scales, including also simultaneous and separate changes in different process parameters.

Building up on results of [1] we present a multiple filter technique (MFT) that detects change points in the rate and variance of point processes on multiple time scales simultaneously. In particular, we use a filtered derivative process and its limit behavior under stationarity. The method also extends to higher order moments.

The separate detection of rate and variance changes requires two techniques: First, rate changes need to be detected, irrespective of potential variance changes. To this end, our approach allows the identification of rate changes in point processes with a certain variability in their lifetimes. Second, the identified rate changes need to be considered when analyzing variance changes. We investigate the empirical properties of our asymptotic MFT method in simulations and apply the MFT to spike trains recorded from auditory cortex of behaving mice, illustrating rate and variability dynamics during the task.
